# Protocol for the 'e-Nudge trial': a randomised controlled trial of electronic feedback to reduce the cardiovascular risk of individuals in general practice [ISRCTN64828380]

**DOI:** 10.1186/1745-6215-7-11

**Published:** 2006-04-28

**Authors:** Tim A Holt, Margaret Thorogood, Frances Griffiths, Stephen Munday

**Affiliations:** 1 Health Services Research Institute, Warwick Medical School, Gibbet Hill Rd, Coventry CV4 7AL, UK; 2 South Warwickshire Primary Care Trust, Westgate House, Market St, Warwick CV34 4DE, UK

## Abstract

**Background:**

Cardiovascular disease (including coronary heart disease and stroke) is a major cause of death and disability in the United Kingdom, and is to a large extent preventable, by lifestyle modification and drug therapy. The recent standardisation of electronic codes for cardiovascular risk variables through the United Kingdom's new General Practice contract provides an opportunity for the application of risk algorithms to identify high risk individuals. This randomised controlled trial will test the benefits of an automated system of alert messages and practice searches to identify those at highest risk of cardiovascular disease in primary care databases.

**Design:**

Patients over 50 years old in practice databases will be randomised to the intervention group that will receive the alert messages and searches, and a control group who will continue to receive usual care. In addition to those at high estimated risk, potentially high risk patients will be identified who have insufficient data to allow a risk estimate to be made. Further groups identified will be those with possible undiagnosed diabetes, based either on elevated past recorded blood glucose measurements, or an absence of recent blood glucose measurement in those with established cardiovascular disease.

**Outcome measures:**

The intervention will be applied for two years, and outcome data will be collected for a further year. The primary outcome measure will be the annual rate of cardiovascular events in the intervention and control arms of the study. Secondary measures include the proportion of patients at high estimated cardiovascular risk, the proportion of patients with missing data for a risk estimate, and the proportion with undefined diabetes status at the end of the trial.

## Background

### Primary research question

Can an automated system of electronic feedback (e-Nudge) reduce the incidence of cardiovascular events in high risk patients in general practice, compared to "usual care"?

### Background

A major focus of chronic disease management is the prevention of cardiovascular disease (CVD). An important development occurred in UK primary care in April 2004, with the introduction of the "new General Medical Services Contract" (nGMS) [1]. This involved the establishment of registers for a number of conditions relevant to CVD prevention, and the resulting standardisation of electronic record keeping has made the data potentially useful for research [2]. The Quality and Outcomes Framework (QOF) of the nGMS specifies targets for blood pressure, serum cholesterol levels, and smoking cessation advice for patients with hypertension, diabetes, or established CVD. Whilst not included in the QOF, the Coronary Heart Disease (CHD) National Service Framework (NSF) [3] also recommends the systematic identification of patients at high risk of CHD but who are not yet displaying any symptoms.

For many patients, the need for preventive treatment is clear, for example through a diagnosis of CVD or diabetes, but for others, the overall cardiovascular risk should be taken into account when determining the need for treatment of hypertension or hypercholesterolaemia. This strategy is strongly supported by the recently published Joint British Societies guidelines on prevention of CVD in clinical practice [4]. This project will assess the effectiveness of targeting patients who are the most likely to benefit from risk factor modification on the basis of their absolute risk of cardiovascular events.

### Changing clinical behaviour through electronic reminders

Despite increasing use of electronic reminders and alert messages, there are relatively few controlled trials that demonstrate their ability to modify clinical behaviour, and none so far carried out in the UK under the new General Practice contract. Published literature is largely concerned with the following uses of these tools:

• to increase physician or nurse adherence to guidelines on best practice in the clinical environment [5], including the use of drug therapy [6]

• to increase the uptake of vaccinations [7-11]

• to promote other preventive health care activities, by triggering opportunistic interventions including screening [12], monitoring [13,14], diagnostic tests [15], and lifestyle counselling [16,17]

• to increase the cost-effectiveness of health care, by avoiding duplication, facilitating communication between members of the health care team [18], and reducing the need for recall of patients through increased use of opportunistic activities during consultations

Of these, the most successful area is vaccination uptake, where a number of studies have demonstrated benefit [7-10], and in the avoidance of prescribing errors, where alerts have been shown to be effective in decreasing the ordering and administration of contraindicated drugs, for instance due to renal insufficiency [19].

Results in other areas have been mixed [20], and may depend on the response of the clinician to the alert message, which must therefore be appropriately designed [21]. In a United States outpatient clinic setting, Tierney et al [22] tested the effects of a system of electronic 'suggestions' for cardiac care patients through a randomised controlled trial, and failed to demonstrate any control-intervention differences in quality of life, medication compliance, health care utilisation, costs, or satisfaction with care. The intervention had no effect on physicians' adherence to the care suggestions. However in Italy, electronic reminders have been shown to be effective in modifying prescribing behaviour. Filippi et al [23] investigated the effects of computerised reminders plus a letter describing the beneficial effects of anti-platelet therapy (intervention group) with the letter alone (controls) among 300 Italian general practitioners randomised to each group. The number of treated patients was significantly raised in the patients of the intervention group (OR 1.99, 95% CI 1.79 – 2.22).

In Scotland, the CARDIA (Computerised Automated Risk Detection Intervention and Advice) program [24] serves practices throughout Angus using a similar system of database integration as that proposed in this e-Nudge study. CARDIA interrogates the electronic health record (EHR), which uses information from both primary and secondary care sources. CARDIA targets resources by examining the practices' EHRs, identifying patients with existing cardiovascular disease (or those at high risk of it based on a Framingham calculation), and assesses the adequacy of care (e.g. drug therapy) in individual patients. However the effectiveness of this program has not been formally tested in a clinical trial.

In secondary care, Lilford et al [25] have described (but not evaluated) a system of electronic reminders for use in the antenatal clinic. This system supplies action suggestions during the antenatal booking interview, as a complement to individual clinical judgement. Eighty-two different suggestions were included in the software, and on average 1.5 of these were generated in an individual history. The authors emphasise the potential for such systems to be adapted to the resources and preferences of different hospitals.

### Controlled studies similar to the e-Nudge trial

One randomised controlled trial in primary care [26] has assessed the effectiveness of electronic feedback using off-line data analysis followed by a flag in the electronic health record. Randomisation was at the health professional level. The outcome was the proportion of patients under the care of each professional still eligible for an alert a month later. This design is in some ways similar to this e-Nudge trial, and the result was positive, but it took place in the USA and only involved one cycle of data analysis with follow-up one month later. In secondary care, the effectiveness of a similar intervention aimed at clinicians caring for hospital inpatients at risk of deep vein thrombosis (DVT) was more dramatically demonstrated [27]. In this case randomisation was at the individual patient level and the outcome was the actual development of DVT. The intervention group patients were found to have a 40% reduced rate of thrombosis compared with controls. A similarly-designed study of electronic reminders for the improved care of patients with HIV infection achieved a significant reduction in hospitalisation in the intervention group [28].

Mitchell et al [29] used information extracted from Scottish general practices to target care towards those aged 65–79 years most in need of intervention for their blood pressure. Information was extracted annually, and 54 practices were cluster-randomised into three groups: those receiving feedback of information identifying patients with uncontrolled blood pressure, those receiving the same feedback but including patients' estimated absolute cardiovascular risk, and control practices receiving no feedback. Whilst reductions in the proportion of patients with controlled blood pressure were seen, the results were compromised by difficulties in stratification according to practice characteristics (resulting in an excess of controls that were training practices, and having a hypertension recall system).

Evidence published to date suggests that the benefits of electronic reminders are context-dependent, relying not only on the area of care involved, but also on organisational parameters, clinical targets, and medicolegal implications. A Veterans Health Administration study [30] demonstrated significant variation in the implementation of electronic reminders including their greater use for conditions associated with performance measures. Agarwal et al [31], in a study of 15 different computerised reminders found that while overall adherence was high, there is significant variation by clinic, individual clinician and individual reminder. For instance, the hepatitis C risk assessment reminder was found to have the highest overall adherence rate (95.9%) and the tobacco use cessation had the lowest adherence rate (62.9%). Dickey et al [32] have reviewed the literature on a range of office based tools for improving behavioural change counselling in primary care. This included all types of tool, including electronic reminders. They found that no one type of tool or method of teamwork was consistently more effective than another, and identified the need for more high quality research, particularly in the area of health risk assessment and electronic reminder systems.

There is therefore mixed evidence supporting the effectiveness of electronic reminders and a need to confirm their ability to modify clinical behaviour in the particular context of UK primary care under the new GMS Contract.

### Overview of study design

This is a randomised controlled trial to test the effect of an automated electronic feedback system on CVD prevention in general practice. The practice populations over the age of 50 years will be randomised into two groups: "intervention" and "control". Intervention patients currently belonging to one of the high risk search groups described below will have alert messages appear on the screen when their electronic notes are opened. We will also apply an electronic search protocol every eight weeks to both groups throughout the study, to produce continually updated lists of potentially high risk patients for cardiovascular events. For the intervention group the patients on these lists will be revealed to the practice. The clinical software company EMIS, who serve the majority of practices in Warwickshire and Coventry, have programmed their software to produce the alerts and the eight-weekly lists for intervention patients. This "intervention" involves the feedback to practice teams to identify patients who are currently at high estimated risk, patients whose data is incomplete (who may benefit from updated measurements of cholesterol, blood glucose, blood pressure or recording of smoking status) and those who may have undiagnosed diabetes, through the alert messages and the eight-weekly lists. The control group will receive the usual care provided under the nGMS contract. No information will be withheld from the clinicians regarding control patients, the only difference will be the absence of reminders to draw their attention to the information. The practice teams themselves will decide on any changes in treatment in consultation with individual patients in both arms of the study, allowing care to remain tailored at the clinician-patient level. Outcomes will include the number of cardiovascular events and the number of high risk patients in the two populations (defined by inclusion on the eight-weekly search results). The design of the search protocol and the justification for the thresholds are described in the appendix.

## Methods

### Recruitment

Up to twenty-six general practices in Coventry and Warwickshire who use EMIS LV software will be invited to participate in the trial. Dr Tim Holt will visit each practice to explain the trial and gain written consent from the general practitioners.

### Randomisation

Participating practice patients over 50 years of age will be randomised into two groups – "intervention" and "control." Patients will be consistently allocated to these groups throughout the study using an electronic technique that is concealed to all researchers and practitioners involved. This process will occur electronically during each search, so that those who join the practice during the study will be randomised automatically as soon as they are first provided with electronic notes as a fully registered patient. Temporary residents are not included in the study.

### Applying the search strategy

**Figure 1:**
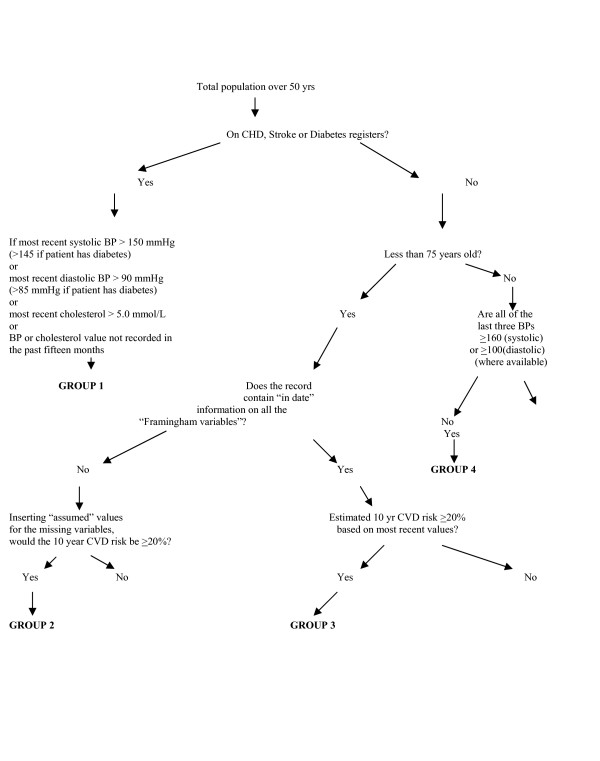
Search algorithm to identify those most likely to benefit from cardiovascular prevention based on recent risk variable values. Definitions for terms in inverted commas are given in the appendix along with justification of thresholds and search protocol.

**Figure 2:**
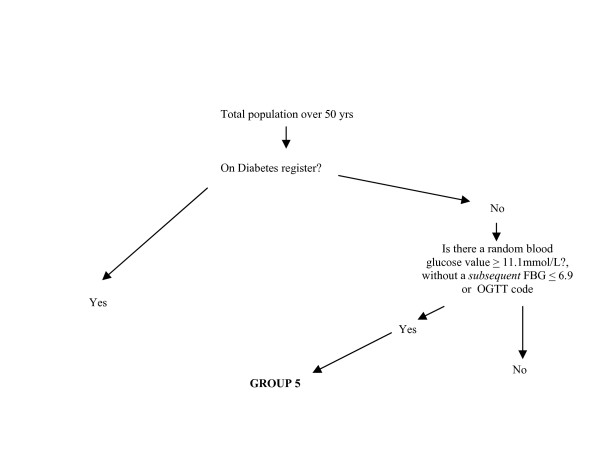
Identification of Group 5.

**Figure 3:**
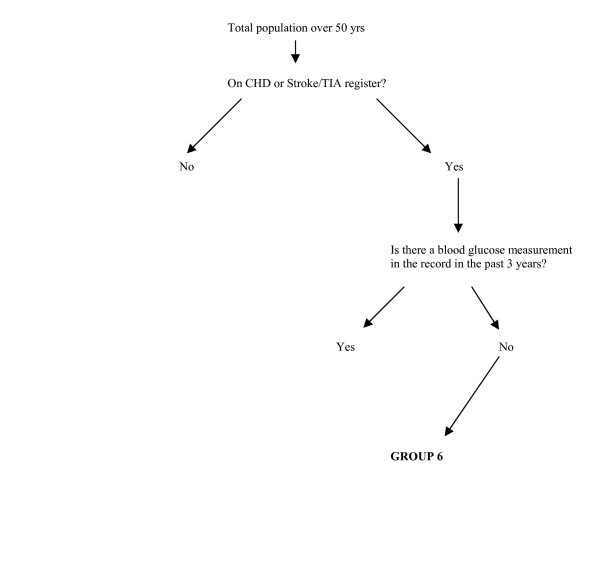
Identification of Group 6.

Alerts will be created automatically using patient information that is updated in real time and the search protocol described in Figures 1, 2, 3. For the eight-weekly lists we will apply the same search protocol to the databases of participating practices. This will produce lists for each practice of the high modifiable risk patients in the intervention arm of the study on the day of the search. The groups identified can be summarised as:

GROUP 1: Patients of all ages with existing cardiovascular disease or diabetes, whose blood pressure or cholesterol level is outside the QOF target range at the last estimation, or no "in date" level is recorded.

GROUP 2: Patients who are not known to have cardiovascular disease or diabetes, are *under *75 yrs old, and whose risk profile is incomplete – more information is required to perform a risk estimate – but whose cardiovascular risk would be greater than 20% if the "assumed" values of the missing factors are used (see definition in appendix).

GROUP 3: Patients who are not known to have cardiovascular disease or diabetes, are *under *75 yrs old, and whose most recent risk variable values indicate that their risk level is raised.

GROUP 4: Patients who are not known to have cardiovascular disease or diabetes, are *greater than *75 yrs old and who have persistently elevated blood pressure based on the three most recent consecutive readings.

GROUP 5: Patients with possible undiagnosed diabetes on the basis of at least one previous high blood glucose record.

GROUP 6: Patients with CVD but not diabetes, who have not had a blood glucose measurement in the past three years.

**Figure 4:**
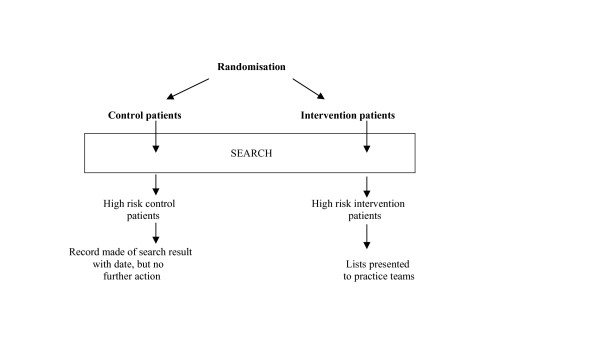
Eight-weekly searches on practice databases.

Information on the "intervention" patients identified at each search is revealed to the practices. Information on the control patients including the number identified will be saved but no action will be triggered (Figure 4).

### Intervention – the "e-Nudge"

The e-Nudge is an automated feedback system that examines information already contained in practice databases to help practice teams target preventive interventions towards the individuals most likely to benefit. At the same time, the e-Nudge identifies clinically important missing risk variable values and patients with possible undiagnosed diabetes. Designed to run as a series of updated alert messages and searches that use most recent risk variable values, it is able to track practice populations over time as patients enter and leave the area, grow older, and enter practice disease registers, such as those for diabetes, CHD, or stroke. It recognises that risk profiles are dynamic, and that "one-off" estimates of risk in individuals are liable to become outdated [33].

The alert messages will arise automatically through EMIS software when a high risk patient's notes are opened, and are continuously updated in real time. To identify patients who may not present to the practice, electronic searches will be undertaken every eight weeks. The purpose of both alerts and the lists is to trigger awareness of individual patients' risk within the practice team, and not to dictate specific treatments. The "e-Nudge" is therefore simply the feedback of this information. The resulting action is at the discretion of the practice team, and can be tailored both to the time available, and to the needs and preferences of the individual patient in the context of the clinician's broader knowledge of co-morbidity, current medication, and past response to treatment. The practice teams will have the following notifications for intervention patients identified in the searches.

**1 The eight-weekly search result is presented to a nominated member of the primary care team **under the six group headings.

**2 Alert messages **are displayed automatically on the computer screen each time the patients' electronic notes are opened. These are triggered for those identified in any of the groups:

• Group 1 patients: ***This CHD/Stroke/Diabetes (state which) patient's (BP) or (serum cholesterol) level (specify which) is out of the target range***.

• Group 2 patients: ***This patient may be at high cardiovascular risk, but values for the following risk variables were either missing or out of date: (specify which variables)*.**

• Group 3 patients: ***This patient's estimated cardiovascular risk may be elevated, based on the most recent risk variable values.(State assumptions made)***

• Group 4 patients: ***This patient's blood pressure is persistently elevated based on three consecutive values*.**

• Group 5 patients: ***This patient may have undiagnosed diabetes, based on a previous raised blood glucose level ≥ 11.1 mmol/L***.

• Group 6 patients: ***This CHD/Stroke patient (state which) has no recorded blood glucose measurement in the past three years***.

### Control condition

Control patients at high estimated risk will be identified but the practice teams will not be provided with these extra reminders, although the team will have access to all the clinical information used to assess risk status. Control patients will continue to receive the usual care provided by current general practice under the nGMS contract. Some practices have started to use alerts for CVD or Diabetes patients who are out of the nGMS blood pressure and cholesterol targets since this study was conceived. Where this is now 'usual care,' this part of the intervention (Group 1 alerts) will not be withheld from the control patients, but the rest of the e-Nudge (including identification on the eight-weekly searches) will be. The standard of care is high in the study locality [South Warwickshire Primary Care Trust, QOF data on file], providing a suitable environment to test the e-Nudge. If the study shows a positive effect, this will demonstrate that even good care can be improved, and it is anticipated that the tool will be even more effective in environments where care is of a lower standard.

### Ethical approval

The trial has been developed in accordance with the Declaration of Helsinki, and approved by Warwickshire Local Research Ethics Committee (Ref: 05/Q2803/85).

### Outcome analysis

The searches and alerts will continue for a period of two years, at the end of which the data will be examined. We will continue to collect and analyse data on the primary and secondary outcomes of the study for a further year after this. Outcomes will be measured using searches on practice databases. Analysis will be undertaken on an "Intention To Treat" basis within practices. Practices that withdraw will have their data censored from the date of withdrawal from the trial.

### Primary outcome

Difference in the annual incidence rate of cardiovascular events (see definition in the appendix) in the intervention and control populations during the two years of the study, and for a third year following the end of the e-Nudge intervention.

### Secondary outcomes

• Difference in the proportion of high risk patients (Groups 1, 3 and 4) identified in the control and intervention populations averaged over the last three searches in the two year intervention period, and in the third year following the end of the intervention.

• Difference in the proportion of patients in each population identified with missing data (Groups 2 and 6) averaged over the last three searches in the two year intervention period, and in the third year following the end of the intervention.

• Difference in the proportion of patients with undefined diabetes status (i.e. raised blood glucose levels with no diagnosis of diabetes and no FBG or OGTT results to confirm status) (Group 5) in the intervention and control populations averaged over the last three searches in the two year intervention period, and in the third year following the end of the intervention.

### Statistical analysis

Analysis of the data will be carried out in STATA. The principle analyses will be on an intention-to-treat basis and will be performed using the CONSORT guidelines (2001) [34].

### Data monitoring committee

Outcomes will be assessed annually during the study by an independent data monitoring committee, who will inform the trial investigators if the trial should terminate early on ethical grounds due to a 20% difference in mortality or morbidity between the intervention and control groups.

### Data quality assurance measures

We will examine the cause of death of every patient in the practices over age 50 years who dies during the study, to ensure that all cardiovascular deaths are recorded appropriately in searchable form prior to outcome data extraction. Any patient recorded as having more than one cardiovascular event during the study will have their clinical record examined, to identify patients who have had the same event recorded twice (which may happen when a consultation for a stroke, TIA or myocardial infarction is mistakenly labelled as a "new episode" rather than a "review"). This process will be carried out both on controls and intervention patients. In addition, we will examine the notes of any patient who has a record of an event dated within 4 months of registration with a practice, in case this event occurred in the past but was incorrectly dated when the patient registered.

### Sample size calculation

#### Event rates

Our study defines a cardiovascular event as a new diagnosis of CVD, a new myocardial infarction, a new stroke, a new transient ischaemic attack, or sudden death from CVD. A new stroke in someone with a previous stroke will count as a new event. An acute myocardial infarction in a patient previously diagnosed with angina will be recorded as a new event, but a new onset of angina in a patient who already had a diagnosis of acute myocardial infarction might not be recorded as a new diagnosis, as the patient will already be on the CHD register.

The British Heart Foundation [35] has compiled an estimate of the number of cardiac events in the UK population in 2002 from several available data sources. The number of myocardial infarctions (all ages) was estimated to be 268,000, while the number of new cases of angina (all ages) was estimated to be 338,000

The UK population was 59,321,700 in 2002 [Sources: Office for National Statistics, General Register Office for Scotland, Northern Ireland Statistics and Research Agency], so estimated incidence rates *for coronary heart disease *are

**Incidence of myocardial infarction **451.77 per 100,000

**Incidence of new case of angina **569.77 per 100,000

For *cerebrovascular disease*, the OXVASC study [36] provides a local source of information drawn from an Oxfordshire population. The incidence rates were:

**Incidence of stroke **187 per 100,000

**Incidence of TIA **51 per 100,000

Therefore

**Incidence of all cardiovascular events 1260 per 100,000**

#### Clinical significance

We aim to demonstrate at least a 10% reduction in the cardiovascular event rate. This means that for a positive outcome, the event rate in the intervention population must be ≤90% of the event rate in the control population. We therefore estimated the necessary sample size for this reduction to be detected at the 5% level with 80% power.

#### Estimating population size needed

A Poisson distribution model is appropriate for events that are rare on an individual level, occurring randomly and independently at a constant rate in a population [37].

Assuming a Poisson distribution, the formula for the sample size is:



where:

λ_0 _= the expected incidence of cardiovascular events (i.e. 1260/100,000)

δ = new incidence in the intervention group

*z*_1-*α *_= standardised normal distribution value based on 0.05 significance level

*z*_1-*β *_= standardised normal distribution values for 80% power

*N *= total number of patients required in the study

*Nw *= total number of patients required in the study + 10% to account for practice withdrawal

For 80% power and 0.05 significance level (2-tailed) [38] (see Table 1):

**Table 1:** 

Reduction in incidence (%)	*z*_1-*α*_	*z*_1-*β*_	λ_0_	δ	N	Nw
10	1.96	0.8416	0.0126	0.001260	64133.46	70546.80

The practice population required to detect both statistically and clinically significant changes in the cardiovascular event rate is therefore estimated to be approximately **70,000**, the combined list size of all age groups in participating practices.

## Discussion

We have described the protocol of our trial of an electronic reminder system (the e-Nudge) that aims to change general practitioners' behaviour with respect to patients at risk of CVD. The trial will use routinely collected electronic data to repeatedly flag up high-risk patients and will measure the outcomes in terms of cardiovascular event rates and the risk profile of the over-50 year population. Electronic alert messages are now commonly used in the increasingly integrated software environment of UK primary care, but the evidence to support them is inconclusive. This trial will attempt to provide a more robust evidence base for the use of such tools for preventive care in UK general practice.

Operational issues that arose during the design of this project included those of data quality and software interoperability. Because the coding of clinical data under the nGMS is linked to remunerative targets, a widespread standardisation of Read coding has occurred since 2004 in areas of care related to chronic disease management. Without this development it is doubtful that a trial of this design could be conducted. Despite this, the use of alternative codes within the nGMS contract for data such as blood glucose values made the programming of the search algorithm challenging, particularly as more than one hospital laboratory (which generate these data for practices through electronic links) are involved in the study area. The identification of individual patients' smoking status was designed with current recording practice in mind, and this area of the program was the least secure in terms of accuracy, as it is not always possible to determine from electronic records exactly how long ago an ex-smoker quitted. Participating clinicians are made aware of the limitations of this part of the program so that adjustments can be made based on a knowledge of the patient's actual smoking history.

The e-Nudge Trial is an example of a new model of primary care research. It involves the flow of information out of the databases of participating practices to the practising teams, to then influence clinical behaviour and future data patterns. The search techniques involved include not only the identification of patients according to the presence in their notes of coded data, but a *computation *(using in this case the Framingham CVD algorithm) to define a more complex decision boundary between the high risk and low risk patients in a live database. This approach has become necessary in the light of the most recent guidelines on the prevention of cardiovascular disease [4], which explicitly support the definition of the hypertensive and hyperlipidaemic populations according to overall cardiovascular risk, estimated using both risk algorithms and other information known to the clinician. Such algorithms might lend themselves to future adaptation, by broadening the range of input risk variables, the use of alternative statistical models for the classification of high risk groups, and tailoring to regional populations [33].

The appendices describe the evidence behind the choices made in designing the study including thresholds, assumed values, and definitions.

## Appendices

1. Justification for the thresholds and search protocols used in the study

a) Age group

b) The high CVD risk group (Group 3)

2. Identifying patients with undiagnosed diabetes

3. Screening for type 2 diabetes in populations at risk of CVD

4. Search groups 1, 3 and 4

5. Definitions:

a) "In date"

b) "Framingham variables"

c) "Assumed values"

d) "Cardiovascular event"

### 1. Justification for the thresholds and search protocols used in the study

#### a) Age group

We decided to include in the searches only those patients over 50 yrs, as the prevalence of cardiovascular disease begins to climb steeply at this age [35]. As the main outcome involves a comparison of the effect of the intervention on event rates, this will avoid the dilution of each denominator population by low risk patients.

#### b) The high CVD risk group (Group 3)

The group at high risk of CVD (but who do not already have CHD, Stroke/TIA, or Diabetes) is defined not by a simple combination of diagnostic categories, but as the output of a risk prediction algorithm. The Framingham study data [39] is currently the best available source for patients without CVD under 75 years, and is recommended in the CHD NSF [3] and the 2004 British Hypertension Society Guidelines [40], despite some concern over its applicability to the UK population [41]. We will be using the *most recent values *as inputs for this algorithm. Whilst the recommended approach is to use values prior to treatment with antihypertensive or lipid lowering therapy, our approach is similar to that applied to individuals in existing prediction tools [42,43] that can compare "pre-treatment" with "post-treatment" risk, to emphasise the impact on risk of intervention such as drug therapy and lifestyle modification. We are therefore making no distinction between the estimated risk levels of two patients with identical risk profiles including blood pressure, one of whom is on antihypertensive treatment and the other is not. In fact the treated patient, whilst having a significantly lower cardiovascular risk than before commencing therapy, still has a higher risk (not recognised by our search protocol) than the otherwise similar patient with the same blood pressure not requiring therapy. Despite this limitation, this approach is currently the most effective means of utilising primary care data (where "pre-treatment" blood pressure or lipid levels are often not identifiable), and is very much in keeping with the monitoring process of the QOF, which measures performance according to the most recent values of variables such as blood pressure or serum cholesterol.

### 2. Identifying patients with undiagnosed diabetes

The application of these searches provides an opportunity to identify patients who may have undiagnosed diabetes. Such searches have been shown to include patients absent from diabetes registers with blood glucose measurements above the usual diagnostic threshold of 11.1 mmol/L. For instance, the Diabetes Audit and Research in Tayside Scotland (DARTS) study [44] identified 701 patients with isolated hyperglycaemia in a number of primary and secondary care registers, from a population of 391 274. This figure was 9.2% of the 7596 identified with diabetes. Whilst such patients (particularly if asymptomatic) require further investigation to clarify their diabetes status [45], a number may benefit through earlier detection and treatment if diabetes is confirmed. During pilot work in one local practice, a search identified the following (see Table 2):

**Table 2:** 

Currently registered patients:	12,245
Plasma glucose on record ≥ 11.1 mmol/L but no diagnosis of diabetes	6

Of these six:

1. Four patients had undiagnosed type 2 diabetes later confirmed by fasting blood glucose measurements.

2. One patient had impaired fasting glycaemia (FBG 6.9 mmol/L) and is awaiting further investigation with OGTT to exclude diabetes.

3. One patient had probable steroid induced hyperglycaemia and has had a normal blood glucose value recorded since stopping the steroids.

We are therefore including as part of the regular searches a query to identify such patients, who may have undiagnosed diabetes based on previous raised measurements. Such patients identified in this study will need to have a subsequent non-diabetic fasting blood glucose level (≤6.9 mmol/L) or Oral Glucose Tolerance Test in order that future searches classify them as not having diabetes (see also appendix 5 below). Some of these patients in whom diabetes appears to be refuted by fasting measurements may go on to have further abnormal plasma glucose levels, in which case they will again be identified as possible cases (Group 5) until a further normal fasting glucose level is obtained, or a diagnosis of diabetes is made.

### 3. Screening for type 2 diabetes in populations at risk of CVD

Diabetes UK has issued a position statement on the early identification of people with type 2 diabetes [46]. Among other groups, this document identifies people with ischaemic heart disease, cerebrovascular disease, peripheral vascular disease or hypertension as high risk groups justifying screening, with a screening interval of three years. However a reliable and practical screening test has not been established. Whilst fasting plasma glucose estimation is significantly more specific than random plasma glucose estimation, it is less practical. In addition to the detection of possibly undiagnosed patients described above, we have therefore designed the study to encourage blood glucose testing at least every three years in groups who either have, or who are at high risk of CVD. These tests can be carried out during the routine blood checks that patients receive for monitoring of lipid lowering or anti-hypertensive therapies. Therefore negative diabetes status will only be assumed if the patient is not on the Diabetes register *and *a normal plasma glucose level (random or fasting) is present in the record within the three years prior to the search. We will be allowing the follow up of patients with borderline plasma glucose levels to remain at the discretion of the practices. (The recently published Joint British Societies guidelines on prevention of cardiovascular disease in clinical practice (JBS 2), clarifies currently recommended practice in this area for the first time [4].) This study may be able to determine whether this approach is useful as a means of detecting type 2 diabetes earlier in these groups, given its practicality and low cost. Practices are at liberty to use more specific screening tests on any individual whom they feel justifies it.

### 4. Search groups 1, 3 and 4

The Group 1 patients are identified on the basis of thresholds used as audit targets in the nGMS contract for secondary prevention. Whilst these treatment targets are essentially arbitrary [47], they have been selected through extensive discussions between the Department of Health and expert advisory bodies. Following advice in the National Service Framework for Diabetes [48] and supported by the 2004 BHS guidelines [40] and JBS 2 [4], the nGMS QOF recommends that patients with diabetes are treated as if they already have cardiovascular disease in terms of cholesterol and blood pressure control. The latter in fact requires tighter target levels than for patients with CVD alone. For this reason they will similarly be regarded as secondary prevention patients in this study.

For primary prevention (Group 3 and Group 4), the British Hypertension Society Guidelines (2004) recommend a 10-year risk of developing cardiovascular disease of ≥20% as a threshold for treatment of grade I hypertension with antihypertensive drugs, or lipid lowering therapy in all groups at this risk level up to the age of 80 yrs [40]. However, the Framingham algorithm is not designed to be used in patients over 75 years of age, and the CHD NSF [3] recommends that the systematic identification of new primary prevention candidates (particularly for lipid lowering therapy) should stop at age 74 years. However, older hypertensive patients benefit from blood pressure reduction and the identification of patients with grade II hypertension or higher, based on serially elevated blood pressure measurements can therefore be justified above this age limit. Whilst it might be justifiable to reduce this threshold (for instance to identify older patients with grade I rather than grade II hypertension), this would involve identifying potentially large numbers of patients whose need for treatment was not as clear, adding considerably to the workload involved.

### 5. Definitions

5a "**In date**" means:

1. A blood pressure reading within the last fifteen months for patients who have CHD/Stroke/TIA or Diabetes, otherwise three years.

2. A blood glucose level within the last three years (for those without diabetes).

3. A cholesterol level in the last fifteen months for CHD, Stroke/TIA or Diabetes patients, and three years for non-CHD/Stroke/TIA, non-Diabetes patients (applies to possible Group 2 patients, see next section).

5b "**Framingham variables**", in this study means:

1. Age

2. Sex

3. Smoking status (considered positive if record of smoking tobacco at last use of this Read code group, however long ago). A *previously recorded *smoker who has stopped will be considered a non-smoker only if 1 year has elapsed since quitting. Therefore a "smoker" is anyone who has smoked tobacco regularly in the past 1 year.

4. Systolic blood pressure – average of last three "in date" values if available. If there are fewer measurements available, then the average of these is taken.

5. Total serum cholesterol at most recent measurement, if "in date"

6. Serum HDL cholesterol – as for total cholesterol

7. Left Ventricular Hypertrophy status – assume negative unless there is any positive electronic record of LVH.

8. Diabetes status, according to whether or not the patient is on the Diabetes register. However, as discussed above, this depends on the quality of such registers. If a primary prevention patient less than 75 yrs does not have a diagnosis of diabetes, but there is no blood glucose level "in date" (i.e. in the past three years), then the risk algorithm will base the risk calculation on an assumption of *positive *Diabetes status, and if the risk level is then high, the practice will be notified with this assumption stated, as a Group 2 Alert message. If a patient (this time including those above 75 yrs) is not on the Diabetes register but there is a record of a blood glucose level greater than or equal to 11.1 mmol/L, then the practices will be notified for clarification, regardless of the patient's CHD/Stroke status or calculated risk level as a Group 5 patient. The matter can be clarified by the practice teams if they wish, by organising a Fasting Blood Glucose (FBG) or Oral Glucose Tolerance Test (OGTT). A FBG ≤6.9 mmol/L or OGTT code following (at a later date to) the high random blood glucose level will mean that the patient is no longer in Group 5 (but may re-enter it if further raised blood glucose levels occur). The FBG or OGTT must be clearly recorded electronically by the practices using appropriate codes (to distinguish fasting values from random blood glucose values), or the patient will continue to be flagged up in subsequent searches. If, despite a normal FBG result or OGTT, a *further *raised random value subsequently occurs (≥ 11.1 mmol/L) then once again the program will question whether or not the patient has diabetes by including them in Group 5, until a *further *FBG ≤ 6.9 or OGTT code is recorded, or the patient is diagnosed and added to the Diabetes register.

5c "**Assumed values**" for the missing variables means:

1. For systolic blood pressure: Male 135 mmHg, Female 132 mmHg

2. For total serum cholesterol: Male 5.7 mmol/L, Female 6.2 mmol/L

3. For HDL cholesterol: Male 1.4 mmol/L, Female 1.7 mmol/L

4. For diabetes: positive status.

5. For smoking status: non-smoker.

These blood pressure and cholesterol thresholds are the approximate median or mean values in the 50–74 year age group taken from the Health Survey for England 2003 [49].

5d A **"cardiovascular event" **is defined as:

1. A new diagnosis of cardiovascular disease (i.e. entry onto the CHD or Stroke/TIA registers)

2. A new stroke or transient ischaemic attack (TIA) (whether or not already on the Stroke register)

3. A new myocardial infarction (whether or not already on the CHD register).

4. Sudden death from cardiovascular disease.

## Abbreviations

CVD Cardiovascular disease

CHD Coronary heart disease

TIA Transient ischaemic attack

DVT Deep vein thrombosis

nGMS The new General Medical Services contract in UK primary care

QOF Quality and Outcomes Framework of the nGMS

BHS British Hypertension Society

JBS 2 The second report of the Joint British Societies on the prevention of cardiovascular disease in clinical practice

EHR Electronic health record

FBG Fasting blood glucose

OGTT Oral glucose tolerance test

LVH Left ventricular hypertrophy

## Competing interests

The author(s) declare that they have no competing interests.

## Authors' contributions

TH is the Principal Investigator and takes responsibility for the day to day running of the trial. MT is the Chief Investigator. MT and FG have assisted in the design of the trial and the development of the protocol. SM has advised on the implementation issues through the local Primary Care Trusts and general practices. All authors have contributed to the drafting of this article.

## References

[B1] http://www.bma.org.uk/ap.nsf/Content/qof06~summclinical.

[B2] Hippisley-Cox J, O'Hanlon S, Coupland C (2004). Association of deprivation, ethnicity, and sex with quality indicators for diabetes: population based survey of 53,000 patients in primary care. BMJ.

[B3] National Service Frameworks (2000). Coronary Heart Disease. Chapter Two: Preventing coronary heart disease in high-risk patients.

[B4] JBS 2 (2005). Joint British Societies' guidelines on prevention of cardiovascular disease in clinical practice. Heart.

[B5] Lobach DF (1996). Electronically distributed, computer-generated, individualized feedback enhances the use of a computerized practice guideline Proc AMIA Annu Fall Symp.

[B6] Kralj B, Iverson D, Hotz K, Ashbury FD (2003). The impact of computerized clinical reminders on physician prescribing behavior: evidence from community oncology practice. Am J Med Qual.

[B7] Weaver FM, Goldstein B, Hammond M (2004). Improving respiratory vaccination rates in veterans with spinal cord injury/disorders: lessons learned. SCI Nurs.

[B8] Kleschen MZ, Holbrook J, Rothbaum AK, Stringer RA, McInerney MJ, Helgerson SD (2000). Improving the pneumococcal immunization rate for patients with diabetes in a managed care population: a simple intervention with a rapid effect. Jt Comm J Qual Improv.

[B9] Tang PC, LaRosa MP, Newcomb C, Gorden SM (1999). Measuring the effects of reminders for outpatient influenza immunizations at the point of clinical opportunity. J Am Med Inform Assoc.

[B10] Hak E, van Essen GA, Stalman WA, de Melker RA (1998). Improving influenza vaccination coverage among high-risk patients: a role for computer-supported prevention strategy?. Fam Pract.

[B11] Lieu TA, Black SB, Ray P, Schwalbe JA, Lewis EM, Lavetter A, Morozumi PA, Shinefield HR (1997). Computer-generated recall letters for underimmunized children: how cost-effective?. Pediatr Infect Dis J.

[B12] Khoury AT, Wan GJ, Niedermaier ON, LeBrun B, Stiebeling B, Roth M, Alexander CM (2001). Improved cholesterol management in coronary heart disease patients enrolled in an HMO. J Healthc Qual.

[B13] Hoch I, Heymann AD, Kurman I, Valinsky LJ, Chodick G, Shalev V (2003). Countrywide computer alerts to community physicians improve potassium testing in patients receiving diuretics. J Am Med Inform Assoc.

[B14] Stewart K, Loftus S, DeLisle S (2003). Prescription of amiodarone through a computerized template that includes both decision support and executive functions improves the monitoring for toxicities AMIA Annu Symp Proc.

[B15] Toth-Pal E, Nilsson GH, Furhoff AK (2004). Clinical effect of computer generated physician reminders in health screening in primary health care – a controlled clinical trial of preventive services among the elderly. Int J Med Inform.

[B16] Intille SS (2004). A new research challenge: persuasive technology to motivate healthy aging. IEEE Trans Inf Technol Biomed.

[B17] Gandhi TK, Sequist TD, Poon EG, Karson AS, Murff H, Fairchild DG, Kuperman GJ, Bates DW (2003). Primary care clinician attitudes towards electronic clinical reminders and clinical practice guidelines AMIA Annu Symp Proc.

[B18] Weiner M, Callahan CM, Tierney WM, Overhage JM, Mamlin B, Dexter PR, McDonald CJ (2003). Using information technology to improve the health care of older adults. Ann Intern Med.

[B19] Galanter WL, Didomenico RJ, Polikaitis A (2005). A trial of automated decision support alerts for contraindicated medications using computerized physician order entry. J Am Med Inform Assoc.

[B20] Yarnall KS, Rimer BK, Hynes D, Watson G, Lyna PR, Woods-Powell CT, Terrenoire J, Barber LT (1998). Computerized prompts for cancer screening in a community health center. J Am Board Fam Pract.

[B21] Schellhase KG, Koepsell TD, Norris TE (2003). Providers' reactions to an automated health maintenance reminder system incorporated into the patient's electronic medical record. J Am Board Fam Pract.

[B22] Tierney WM, Overhage JM, Murray MD, Harris LE, Zhou XH, Eckert GJ, Smith FE, Nienaber N, McDonald CJ, Wolinsky FD (2003). Effects of computerized guidelines for managing heart disease in primary care. Journal of General Internal Medicine.

[B23] Filippi A, Sabatini A, Badioli L, Samani F, Mazzaglia G, Catapano A, Cricelli C (2003). Effects of an automated electronic reminder in changing the antiplatelet drug-prescribing behavior among Italian general practitioners in diabetic patients: an intervention trial. Diabetes Care.

[B24] http://www.eguidelines.co.uk/awards/griffith_awards_oct02.html.

[B25] Lilford RJ, Chard T (1984). The use of a small computer to provide action suggestions in the booking clinic. Nippon Sanka Fujinka Gakkai Zasshi Acta Obstetrica et Gynaecologica Japonica.

[B26] Krall MA, Traunweiser K, Towery W (2004). Effectiveness of an electronic medical record clinical quality alert prepared by off-line data analysis. Medinfo.

[B27] Kucher N, Koo S, Quiroz R, Cooper JM, Paterno MD, Soukonnikov B, Goldhaber SZ (2005). Electronic alerts to prevent venous thromboembolism among hospitalized patients. N Engl J Med.

[B28] Safran C, Rind DM, Davis RB, Ives D, Sands DZ, Currier J, Slack WV, Makadon HJ, Cotton DJ (1995). Guidelines for management of HIV infection with computer-based patient's record. Lancet.

[B29] Mitchell E, Sullivan F, Grimshaw JM, Donnan PT, Watt G (2005). Improving management of hypertension in general practice: a randomised controlled feedback derived from electronic patient data. Br J Gen Pract.

[B30] Fung CH, Woods JN, Asch SM, Glassman P, Doebbeling BN (2004). Variation in implementation and use of computerized clinical reminders in an integrated healthcare system. Am J Manag Care.

[B31] Agrawal A, Mayo-Smith MF (2004). Adherence to computerized clinical reminders in a large healthcare delivery network. Medinfo.

[B32] Dickey LL, Gemson DH, Carney P (1999). Office system interventions supporting primary care-based health behavior change counseling. American Journal of Preventive Medicine.

[B33] Holt TA, Ohno-Machado L (2003). A nationwide adaptive prediction tool for coronary heart disease prevention. Br J Gen Pract.

[B34] Moher D, Schulz K, Altman D (2001). The CONSORT statement revised recommendations for improving the quality of reports of parallel-group randomised trials. Lancet.

[B35] (2004). Coronary heart disease statistics: 2004 Edition.

[B36] Rothwell PM, Coull AJ, Giles MF, Howard SC, Silver LE, Bull LM, Gutnikov SA, Edwards P, Mant D, Sackley CM, Farmer A, Sandercock PA, Dennis MS, Warlow CP, Bamford JM, Anslow P, (Oxford Vascular Study) (2004). Changes in stroke incidence, mortality, case fatality, severity and risk factors in Oxfordshire, UK from 1981 to 2004 (Oxford Vascular Study). Lancet.

[B37] Bland M (2000). An introduction to medical statistics.

[B38] Machin D, Campbell M, Fayers P, Pinol A (1997). Sample size tables for Clinical Trials.

[B39] Anderson KM, Odell PM, Wilson PW, Kannel WB (1991). Cardiovascular disease risk profiles. Am Heart J.

[B40] Williams B, Poulter NR, Brown MJ, Davis M, McInnes GT, Potter JF, Sever PS, Thom S McG (2004). Guidelines for management of hypertension: report of the fourth working party of the British Hypertension Society, 2004-BHS IV. J Hum Hypertens.

[B41] Brindle P, Emberson J, Lampe F, Walker M, Whincup P, Fahey T, Ebrahim S (2003). Predictive accuracy of the Framingham coronary risk score in British men: prospective cohort study. BMJ.

[B42] Hingorani AD, Vallance P (1999). A simple computer program for guiding management of cardiovascular risk factors and prescribing. BMJ.

[B43] Pocock SJ, McCormack V, Gueyffier F, Boutitie F, Fagard RH, Boissel J-P (2001). A score for predicting risk of death from cardiovascular disease in adults with raised blood pressure, based on individual patient data from randomised controlled trials. BMJ.

[B44] Morris AD, Boyle IRD, MacAlpine R, Emslie-Smith A, Jung RT, Newton RW, MacDonald TM, for the DARTS/MEMO Collaboration (1997). The diabetes audit and research in Tayside Scotland (darts) study: electronic record linkage to create a diabetes register. BMJ.

[B45] World Health Organisation (1999). Definition, diagnosis and classification of diabetes mellitus and its complications. Part 1: Diagnosis and classification of diabetes mellitus.

[B46] Diabetes UK (2001). Position statement: Early identification of type 2 diabetes.

[B47] Campbell NC, Murchie P (2004). Treating hypertension with guidelines in general practice. BMJ.

[B48] (2002). National Service Framework for Diabetes: Standards. Clinical care of adults with diabetes.

[B49] Department of Health (2003). Health Survey for England. Risk factors for cardiovascular disease. http://www.dh.gov.uk/assetRoot/04/09/89/11/04098911.pdf.

